# Agreement of Two Physical Behaviour Monitors for Characterising Posture and Stepping in Children Aged 6–12 Years

**DOI:** 10.3390/s23218970

**Published:** 2023-11-04

**Authors:** Esraa Burahmah, Sivaramkumar Shanmugam, Daniel Williams, Ben Stansfield

**Affiliations:** School of Health and Life Sciences, Glasgow Caledonian University, Cowcaddens Road, Glasgow G4 0BA, UK; sivaram.shanmugam@gcu.ac.uk (S.S.); dwilli212@caledonian.ac.uk (D.W.); ben.stansfield@gcu.ac.uk (B.S.)

**Keywords:** children, physical activity, monitoring, agreement, posture, stepping

## Abstract

All new physical behaviour measurement devices should be assessed for compatibility with previous devices. Agreement was assessed between the activPAL4^TM^ and activPAL3^TM^ physical behavior monitors within a laboratory and a multi-day free-living context. Healthy children aged 6–12 years performed standardised (sitting, standing, stepping) (12 min) and non-standardised (6 min) activities in a laboratory and a multi-day (median 3 days) free-living assessment whilst wearing both monitors. Agreement was assessed using Bland–Altman plots, sensitivity, and the positive predictive value (PPV). There were 15 children (7M/8F, 8.4 ± 1.8 years old) recruited. For the laboratory-based standardised activities, sitting time, stepping time, and fast walking/jogging step count were all within ±5% agreement. However, the activPAL4^TM^ standing time was lower (−6.4%) and normal speed walking step count higher (+7.8%) than those of the activPAL3^TM^. For non-standardised activities, a higher step count was recorded by the activPAL4^TM^ (+4.9%). The standardised activity sensitivity and PPV were all >90%, but the non-standardised activity values were lower. For free-living agreement, the standing time was lower (−7.6%) and step count higher (all steps + 2.2%, steps with cadence >100 step/min + 6.6%) for the activPAL4^TM^ than the activPAL3^TM^. This study highlights differences in outcomes as determined by the activPAL4^TM^ and activPAL3^TM^, which should be considered when comparing outcomes between studies.

## 1. Introduction

It has been demonstrated that the performance of adequate levels of physical activity has many health benefits [1]. To gain a full understanding of these benefits, it is necessary to have tools which can measure physical behaviour performance. Only via the measurement of physical behaviour performance (physical activity and sedentary behaviour) can potential interventions to change physical behaviour be assessed. The investigation of an individual’s walking habits and selection of postures throughout the day provides important information on performance which cannot be gained using capability measurement alone.

Different populations have different movement patterns; for example, the very old may walk more slowly and take shorter strides than younger adults and may not engage in extensive activity outside functional walking. Young children undertake more varied movement patterns than adults, with play-like activity where stepping activity might not be considered ‘standard’ in form. As a result, young children’s physical behaviour may pose the greatest challenge to activity classification using body-worn devices.

There are many methods for evaluating physical activity and movement patterns, including both subjective approaches such as questionnaires and objective approaches like the use of body-worn devices. Developments in technology allow the enhancement of body-worn devices, increasing their data collection capacity and reducing device size. ActivPAL^TM^ devices, which are thigh-worn piezoelectric accelerometer-based data loggers, are commonly used body-worn devices. ActivPAL^TM^ (https://www.palt.com/pals/, accessed on 1 October 2023) are set up with proprietary software which allows the characterisation of physical behaviour (time sitting/lying, standing, stepping and step count). PALTechologies Ltd., Glasgow, UK, presented the activPAL4^TM^ device as an enhancement of their activPAL3^TM^. When comparing the outcomes derived from different generations of devices, it is important to understand the agreement between device outcomes to inform a comparison of values. The validity and reliability of the activPAL^TM^ family of devices has been reviewed [2,3], demonstrating high levels of validity (in relation to direct observation) under standardised conditions (original activPAL^TM^ [4]), but lower levels of validity in detecting slow stepping (original activPAL^TM^ [5]) and jogging (original activPAL^TM^ [6]) or in more challenging movement protocols (activPAL4^TM^ [7]). Also, its reliability has been shown to be high (inter-device ICC(1,1) > 0.976 for activPAL4^TM^ [7]); however, between-device agreement has not been studied extensively. Agreement between the original activPAL^TM^ and the activPAL3^TM^ suggested high levels of agreement for standardised activity protocols [8,9] but lower levels for more complex activities, e.g., involving more complex full-body movements or small intermittent stepping activity [8]. The activPAL4^TM^ iteration of the activPAL^TM^ has hardware adaptations compared to the previous activPAL3^TM^. While the manufacturer does not explicitly state that the detection mechanism or software have been changed in the newer version of the monitor, it is important to establish inter-version agreement to inform comparison of outcomes across generations of devices.

This study aimed to establish the agreement between physical behaviour outcomes in the activPAL4^TM^ and activPAL3^TM^ monitors in children aged 6–12 years old. To facilitate this aim, both laboratory-based and free-living data recording were performed.

## 2. Materials and Methods

### 2.1. Participants

For this cross-sectional study, a convenience sample of children (6–12 years old) was recruited in Kuwait City, Kuwait (October 2020–August 2021). The study protocol adhered to the guidelines as set out in the Declaration of Helsinki with approval from the Ethics Committee of Glasgow Caledonian University (ref: HLS/PSWAHS/19/232).

All parents/guardians were provided with an information sheet about the study and required to contact the lead author if they would allow their child to take part in the study. Written informed consent/assent was obtained from all participants. Caregivers were verbally asked about the presence of acute lower limb injuries (excluding bruising and scrapes) or cognitive impairments in their children. If such circumstances were reported, the children were excluded.

### 2.2. The ActivPAL4^TM^ and ActivPAL3^TM^ Monitors

The device documentation indicates that the activPAL3^TM^ (55 × 35 × 6 mm) uses a resolution of 8 bits (range ± 2 g) compared to 10 bits (range ± 4 g) for the activPAL4^TM^ (45 × 25 × 5 mm), suggesting a modification to the hardware and to the data processing algorithm between devices.

**Figure 1:**
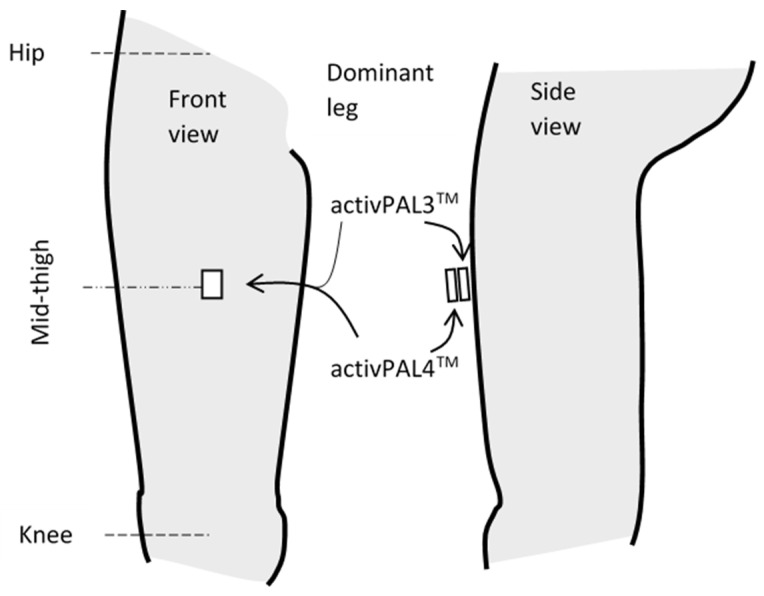
Location of the activPAL4^TM^ and activPAL3^TM^ on the thigh.

Nine activPAL4^TM^ and three activPAL3^TM^ devices were used across participants (software version PALAnalysis v8.10.8.32, minimum non-upright period and upright period 2 s [10], 20 Hz frequency of data collection). The activPAL3^TM^ was placed on the front of the mid-thigh of the dominant leg (Figure 1) with the activPAL4^TM^ piggybacked on top, both secured with hypoallergenic tape. Random allocation of the device order was unsuccessful due to the larger size of the activPAL3^TM^ compared to the activPAL4^TM^ (micro). The activPAL4^TM^ was therefore always placed on top of the activPAL3^TM^.

Event-based outcomes from the activPAL^TM^s were extracted using the VANE algorithm within the PALAnalysis v8.10.8.32 software for both devices. From the PALAnalysis software, activity was divided into events (sitting/lying, standing, stepping) with the associated duration to the nearest 0.1 s. Stepping was characterised as strides (equivalent to two steps) within the stepping time.

### 2.3. Data Collection

The data collection consisted of two parts: a laboratory-based protocol and a free-living multi-day data collection. The laboratory-based protocol was used to provide evidence of agreement for the particular activity types under observation. The free-living multi-day data collection provided evidence of agreement within individuals for typically used daily measures of physical activity and sedentary behaviour.

#### 2.3.1. Laboratory-Based Protocol

The laboratory-based protocol consisted of standardised and non-standardised activities (Table 1) performed within a room approximately 10 m × 5 m in size. Participants were allowed to move freely around the room as they wished within the constraints of the particular activity being performed. This protocol was designed specifically for this study. The standardised activities consisted of defined periods of sitting, standing (without stepping), and stepping activity. The non-standardised activities provided the opportunity for the participants to engage in a range of stepping patterns.

For each activity, participants were asked verbally to commence the activity as specified. For the standardised walking activities, participants were instructed to ‘walk at your preferred speed’ and ‘run/jog or walk at a fast speed’. For the non-standardised activities, participants were given verbal instructions on how to complete the task and given general encouragement to maintain engagement throughout the allocated time period. The elements of the laboratory-based protocol were selected to provide evidence of agreement for clearly defined postures and stepping as well as stepping activity with movement patterns of a more self-selected nature.

#### 2.3.2. Free-Living Multi-Day Protocol

Participants were asked to wear both monitors for up to 3 full days, keeping the monitors in place on the thigh 24 h per day. A minimum of 1 full 24 h data recording period was accepted. The participants wore the same monitors for the laboratory-based and free-living protocol. The activity profiles (activPAL^TM^ proprietary spiral plots) were visually inspected to identify any periods where the monitors had not been worn.

### 2.4. Data Analysis

Group-level amounts of outcomes were characterised descriptively across participants (mean ± SD). The data were manipulated and outcomes derived using the Excel software (Version 2019 MSO 16.0.10402.20023, Microsoft).

#### 2.4.1. Laboratory-Based Protocol

The timings of the activities were recorded in a video-based record. The start and end of the standardised activities were determined, as well as the start and end of the walking periods within the standardised activities. Additionally, the start and end points of the non-standardised activities were recorded in the video-based record. The records of the activPALs were synchronised with the video-based record via the timing of the computer used to initialise the activPALs and the video-recording device. Comparisons of outcomes between the monitors were made based on activity state (sitting, standing, stepping) and step count. Agreement in the activity state was characterised across each set of activities, i.e., across all standardised activities and then all non-standardised activities. Agreement in the step count was performed for the normal and fast walking periods separately and then the entirety of the non-standardised activities.

Event-based outcomes from the activPALs were interpolated to gain a continuous record of posture and stepping activity (sitting/lying, standing (without stepping), stepping) for every 0.1 s. The total number of steps (strides × 2) was determined for the preferred (normal) speed of walking, fast walking/jogging, and for the non-standardised activities.

For the laboratory-based data analysis, agreement was calculated for the amount of time in a posture and stepping for the standardised activities and non-standardised activities. Additionally, across all 10 ths of a second, the agreement, sensitivity, specificity, positive predictive value (PPV), and negative predictive value (NPV) were determined.

#### 2.4.2. Free-Living Multi-Day Protocol

Only full 24 h periods of successful data recording were used. A 24 h day was decided to start at 4:00 am based on the observation that the majority of participants were awake at midnight and were asleep at 4:00 am. The mean daily value was used per participant across all days of data. For the amount of sitting/lying, standing, and stepping time, number of sit-to-stand transitions, all step counts, and steps taken at above a cadence of 100 steps per minute of agreement were determined.

#### 2.4.3. Agreement—Amount of Activity

Agreement was assessed for posture and step count using Bland–Altman plots (Equations (1) and (2)) [11] with limits of agreement (LoA, ±1.96 × SD) quantifying measurement variability.

(1) $$y\;axis=\frac{activPAL4\;measure-activPAL3\;measure}{activPAL3\;measure}\times 100$$

(2) $$x\;axis=\frac{activPAL4\;measure+activPAL3\;measure}{2}$$

To assess whether the agreement between devices was acceptable, an a priori upper limit of 5% difference was chosen. This was chosen pragmatically, to highlight substantial differences that were unlikely to be due to minor elements of device manufacture/operation (e.g., synchronization or time-based drift) and were related to important differences in outcomes.

#### 2.4.4. Agreement—Across Time

The percentage of time for which there was agreement was calculated across all relevant 10 ths of a second (Equation (3)).

(3) $$\%\;of\;agreement=\frac{number\;of\;instances\;where\;activPAL4\;posture=activPAL3\;posture}{number\;of\;instances}\times 100$$

Sensitivity, specificity, positive predictive value (PPV), and negative predictive value (NPV) were also calculated [12]. For the time that the activPAL3^TM^ recorded a certain posture, sensitivity was determined as the proportion of time for which the activPAL4^TM^ agreed (Equation (4)):

(4) $$Sensitivity=\frac{number\;of\;instances\;where\;posture\;classifications\;of\;activPAL4\;and\;those\;of\;activPAL3=A}{number\;of\;instances\;where\;activPAL3\;posture\;classification=A}$$

For the time that the activPAL3^TM^ did not record a specific posture, the specificity was defined as the proportion of that time for which the activPAL4^TM^ also did not record that posture (Equation (5)):

(5) $$Specificity=\frac{number\;of\;instances\;where\;posture\;classifications\;of\;activPAL4\;and\;those\;of\;activPAL3\neq A}{number\;of\;instances\;where\;activPAL3\;posture\;classification\neq A}$$

Within the time that the activPAL4^TM^ determined a particular posture, the PPV characterised the proportion of time for which the activPAL3^TM^ agreed (Equation (6)):

(6) $$PPV=\frac{number\;of\;instances\;where\;posture\;classifications\;of\;activPAL4\;and\;those\;of\;activPAL3=A}{number\;of\;instances\;where\;activPAL4\;posture\;classification=A}$$

For the time that the activPAL4^TM^ did not record a specific posture, the NPV was defined as the proportion of that time that the activPAL3^TM^ also did not record that posture (Equation (7)):

(7) $$NPV=\frac{number\;of\;instances\;where\;posture\;classifications\;of\;activPAL4\;and\;those\;of\;activPAL3\neq A}{number\;of\;instances\;where\;activPAL4\;posture\;classification\neq A}$$

Based on the expectation of the outcomes being the same, values of agreement, sensitivity, specificity, PPV, and NPV were classified as low (<80%), moderate (80–90%), or high (>90%).

#### 2.4.5. Further Results Exploration

Where specific high levels of disagreement between monitor outcomes were highlighted for individuals, further investigation was planned with outliers removed to establish the underlying agreement between monitors.

## 3. Results

Of the 15 participants who were recruited to the study, only 13 (6M/7F, mean age 8.3 years old, SD 1.8, range 6.3–12.2) successfully completed the laboratory-based protocol due to a failure in video recording in one case and failure to collect data for one activPAL4^TM^. The sample size was relatively small. However, the laboratory-based protocol included various tasks that tested the activPAL^TM^’s performance in detecting posture and stepping.

Variation in the combination of specific activPAL4^TM^ and activPAL3^TM^ devices resulted in two of the activPAL3^TM^s being paired with four different activPAL4^TM^s, and one with five different activPAL4^TM^s.

### 3.1. Laboratory-Based Agreement

Sometimes, there were delays in response to instructions, and there was a variety of different interpretations of walking at normal speed or faster speed, or jogging.

Sitting time, stepping time, and fast walking/jogging steps during the standardised activities all demonstrated levels of agreement within the a priori set limit of ±5% (Table 2). However, a lower standing time was measured by the activPAL4^TM^ than the activPAL3^TM^ (−6.4%), with more steps recorded during normal-speed walking (+7.8%). Standing time and normal walking step count had notably wider limits of agreement than other outcomes.

Due to the nature of the non-standardised activities, a relatively low amount of sitting was recorded (23.0 ± 13.6 s for activPAL4^TM^). This resulted in large percentage mean differences for only small time differences. The most extreme case was 2.3 s for the activPAL3^TM^ and 9.1 s for the activPAL4^TM^, giving 296% difference. This result would not be relevant to the observation of longer periods of sitting time.

For the non-standardised activities, the activPAL4^TM^ recorded less standing time (−9.8%) and more stepping time (+5.3%) than the activPAL3^TM^. This was accompanied by a higher step count (+4.9%), but this was within the 5% a priori set acceptability threshold.

The agreement plots (Supplementary Data Figure S1) indicated that one participant demonstrated high mean difference between monitor outcomes for both standing time and sitting time. This appeared to be associated with sitting with the thigh not horizontal. Another participant had a particularly high mean difference for the normal-speed walking step count. This participant walked particularly slowly, with continually varying direction of travel. When these two participants’ data were removed from the analysis (resulting in *n* = 11), for the standardised activities, the normal walking speed step difference reduced to +2.2% and the standing time to −0.7% for the activPAL4^TM^ compared to the activPAL3^TM^ (Supplementary Data Table S1, Figure S2). However, for the non-standardised activities, the standing time was still lower for the activPAL4^TM^ (−13.3%) and the step count higher (+7.1%) than for the activPAL3^TM^.

The confusion matrix of time allocation (Table 3) illustrates for the standardised activities that the activPAL3^TM^ standing time was partially redistributed to both the activPAL4^TM^ sitting and stepping time. For the non-standardised activities, 23.5% of the activPAL3^TM^’s standing time was classified by the activPAL4^TM^ as stepping time. Additionally, fractions of the activPAL3^TM^ sitting time (3.4%) and stepping time (5.7%) were classified by the activPAL4^TM^ as standing time.

For the standardised activities, the agreement, sensitivity, specificity, PPV, and NPV were all high (>90%) between monitors (Table 4). However, the standing time sensitivity demonstrated a lower value than the other outcomes (92.0%), and a wide range (44.2–99.9%).

For non-standardised activities, the agreement between monitors was moderate (88.8%). Notably reduced values compared to the standardized activities were seen for standing sensitivity (75.7%) and stepping specificity (79.8%) with all values of PPV (<90%). These results reinforce observations of different classifications of time between monitors (Table 3) with standing time and stepping time detection showing the largest differences.

### 3.2. Free-Living Multi-Day Agreement

Full 24 h data was available for all 15 participants (7M/8F, mean age 8.4 years old, SD 1.8, range 6.3–12.2). Respectively, 11, 2, and 2 participants recorded 3, 2, and 1 full days. 

Agreement in the sitting/lying and stepping time, the number of sit-to-stand transitions, and all steps were all within the acceptable limits (±5%) (Table 5). However, the standing time of the activPAL4^TM^ was lower (−7.6%) than that of the activPAL3^TM^. A higher step count of steps with a cadence >100 steps/min (+6.6%) was determined by the activPAL4^TM^ compared to the activPAL3^TM^ with wide variation (LLOA − 38.8, ULOA + 51.9%).

During free-living activity, one participant demonstrated a particularly low step count (<1000 per day) for both monitors, with a low cadence and poor agreement between the activPAL4^TM^ and activPAL3^TM^’s step count and posture allocation (Supplementary Data Figure S1). When this outlier was removed (resulting in *n* = 14), there remained a lower standing time allocation (−7.0%) for the activPAL4^TM^ and a higher step count (+3.9%) than the activPAL3^TM^ (Supplementary Data Table S2).

## 4. Discussion

This study aimed to establish the agreement between the activPAL4^TM^ and activPAL3^TM^ for children 6–12 years old under both laboratory and free-living conditions. The device manufacturer indicates that different hardware configurations are used in the two devices, meaning that agreement must be assessed to ensure comparability of outcomes. Overall, the agreement was generally good except for time spent standing (without stepping) (lower in the activPAL4^TM^ than the activPAL3^TM^) and some aspects of the step count (higher for activPAL4^TM^ than the activPAL3^TM^). 

### 4.1. Laboratory-Based Agreement

Under standardised activities, the activPAL4^TM^ determined 6.4% less standing time than the activPAL3^TM^. This was associated with a higher sitting and stepping time. In addition to the increase in stepping time (although small at +1.8%), there was a substantial increase in the number of steps counted during normal walking speed (+7.8%). This suggests that the activPAL4^TM^ may have a slightly different standing time detection algorithm, meaning that more time is characterised as stepping time. It is not clear how the proprietary algorithm for step detection works, but if there is a need to first detect stepping time and only then look for steps within this time, it may be the case that the change in standing time detection has a substantial knock-on effect on the step count.

For one participant, within the standardised activities, there was a substantial difference in the sitting and standing time (Supplementary Data Figure S1). This was associated with the activPAL3^TM^ not recording transitions back to sitting from standing, but classifying continuous standing. This may have been due to the participant sitting with their thigh not horizontal. The proprietary signal processing algorithm may require a different change in the angle of thigh inclination to trigger a stand-to-sit transition in the activPAL4^TM^ compared to the activPAL3^TM^, meaning that sitting with the thigh inclined horizontally may provide different classifications in some people. Examination of the agreement for this participant under free-living conditions revealed only a 0.6% higher sitting time for the activPAL4^TM^ compared to the activPAL3^TM^, suggesting that this difference may have been an artefact of the particular laboratory setup and chair type/height.

Examination of the agreement plots for the non-standardised activity sitting time (Supplementary Figure S1) highlighted how the small time spent in this posture resulted in high percentage differences in some cases. These results could not be generalised to situations where longer sitting times were recorded as the percentage errors would likely be lower. This observation is reinforced when the non-standardised activity outcomes are compared with those of the standardised activities for which the sitting periods were longer.

While not above the a priori limit for acceptability, the number of steps detected by the activPAL4^TM^ was 4.9% higher for the non-standardised activities than the activPAL3^TM^. With the removal of the participant who walked particularly slowly as their normal walking speed (Supplementary Data Table S1), the step count difference in the standardised activities reduced to within ±5%, but the mean difference increased for the non-standardised activities to 7.1%, suggesting that differences in step detection between the monitors mainly occurred during more complex movement patterns.

### 4.2. Free-Living Multi-Day Agreement

It is important to assess the outcomes from the monitors as they might be reported. Free-living activity is typically reported in daily amounts of quantities, e.g., [13]. Any change in hardware or software between two versions of the monitor could have affected the detection of transitions between postures (e.g., sit-to-stand) and also the detection of steps. Changes in the detection of steps could have had a knock-on effect on the timing of standing-to-stepping transitions. Changes in the detection of transition points might also cause differences in bout-based outcomes, e.g., bouts of quiet standing (without stepping), or in the number of bouts of sitting.

The free-living assessment confirmed that the activPAL4^TM^ appeared to record, on average, lower amounts of standing (without stepping) time. The time appeared to be reallocated to both sitting/lying and stepping time with both being higher for the activPAL4^TM^. This shift is also reflected in a slightly higher step count for both all steps and a 6.6% higher step count for steps taken at a cadence > 100 steps/min. This suggests that the activPAL4^TM^ has a slightly different detection of standing time and steps than the activPAL3^TM^. These may be linked if there is a requirement to first determine whether stepping is occurring (i.e., upright, but not standing still) before steps can be identified.

The removal of one participant with <1000 steps/day from the analysis did not substantially change the overall results (Supplementary Data Table S2) with standing time still lower and step counts higher for the activPAL4^TM^ than the activPAL3^TM^.

There was a considerable variation in agreement as characterised by the limits of agreement. This was of particular note for the standing time (%LOA −25.5, 10.3) and steps taken at a cadence greater than 100 steps/min (%LOA −38.87, 51.9). These large ranges of outcomes may reflect sensitivity to particular movement patterns that children routinely perform under free-living conditions.

### 4.3. Limitations

A specific laboratory-based protocol was used in this study. The results are a reflection of the proportion of time spent in each posture category and of the specific activities that the participants were asked to perform. Percentage differences in outcomes must therefore be interpreted in relation to the specific protocol used.

The participants were not always immediately compliant and/or interpreted instructions in their own way, resulting in variations in the timing of activities and speed of performance. It is not possible to say how representative the laboratory activities were of real-world behaviour. However, the activities did allow the participants to use their preferred movement patterns, providing more representative data for comparison of the monitors than might have been gathered from a more rigid protocol.

To achieve a representative sample of free-living data, it has been reported that 4–5 days should be collected for adults and up to 4–9 days for children [14]; however, compliance can be variable [15]. The current study was not aimed at quantifying free-living physical behaviour, but instead assessing agreement. Collecting more data for the current study may have provided greater coverage of the typically used motion patterns to test monitor agreement. However, for most participants (11/15), 3 full days were recorded, providing extensive examples of daily living movement patterns to assess agreement. 

While only a small number of activPAL4^TM^ and activPAL3^TM^ monitors were used in the current study, their inter-device reliability has previously been reported as excellent [6,7], suggesting that the number of monitors was sufficient to avoid reliability issues having an impact on outcomes.

The study used a limited sample size. This could have implications for the generalisability of the findings. As identified within this study, particular individuals used particular movement patterns that appeared to reduce agreement in the device outcomes. Using an increased sample size may have revealed additional movement idiosyncrasies that affected monitor agreement. 

## 5. Conclusions

Agreement between the activPAL4^TM^ and activPAL3^TM^ monitors was assessed under laboratory and free-living conditions. The activPAL4^TM^ generally reported lower amounts of standing time and a higher step count than the activPAL3^TM^ in both laboratory-based and free-living data collections. For individual participants, slow stepping or sitting with the thigh at an inclined angle reduced agreement.

While generally acceptable levels of agreement were evident between the activPAL4^TM^ and activPAL3^TM^, comparison of data should be made cautiously as their outcomes are not identical.

When comparing study outcomes which use different versions of physical behaviour and monitoring devices with different recording parameters, it is important to have confidence that data are equivalent. This study highlights differences in some outcomes as determined by the activPAL4^TM^ and activPAL3^TM^, suggesting that changes in the devices have influenced physical behaviour characterisation. These differences should be considered when comparing outcomes between studies.

## Figures and Tables

**Table 1:** Standardised and non-standardised laboratory activities in order of performance.

Type	Activity Description								
Standardised activities	Sitting	Quiet standing without stepping	Sitting	Walking with preferred stepping speed	Sitting	Fast walking/Jogging	Sitting	Sitting and playing a game on a tablet computer	Sitting and drawing
Duration (mins)	2	1	1	1	1	1	1	2	2
Non-standardised activities	Keep up the balloon			Throwing hoops over a post			Musical chairs		
	Participants were instructed to keep a balloon up in the air by hitting it. During the activity, they could move around as they wished.			Participants stood and threw hoops over a post between 1 and 4 m away. When all hoops had been thrown, the participant retrieved the hoops and then the process was repeated until the end of time.			Participants walked/jogged around two chairs. The chairs were placed facing outwards (~1 m apart). When the music stopped, participants sat as quickly as possible. The process was repeated until the end of the time.		
Duration (mins)	2			2			2		

**Table 2:** Laboratory-based mean outcomes for activPAL4^TM^ and activPAL3^TM^ with Bland–Altman percentage mean differences across standardised and non-standardised activities.

Measure (*n* = 13)	ActivPAL4^TM^ Mean (SD)	ActivPAL3^TM^ Mean (SD)	Percentage Mean Difference (LLOA, ULOA) (%) *
Standardised activities			
Duration (s)			
Sitting	538.5 (6.2)	531.5 (25.1)	1.5 (−8.9, 12.0)
Standing	71.6 (3.7)	80.2 (24.7)	−6.4 (−36.7, 24.0)
Stepping	115.6 (6.0)	114.0 (9.7)	1.8 (−10.4, 14.0)
Step count			
Normal walking	91 (21)	89 (26)	7.8 (−34.3, 50.0)
Fast walking/jogging	120 (16)	122 (17)	−2.0 (−15.6, 11.7)
Non-standardised activities			
Duration (s)			
Sitting **	23.0 (13.6)	21.6 (14.3)	29.3 (−130.0, 188.5)
Standing	101.3 (36.2)	112.8 (40.2)	−9.8 (−33.0, 13.3)
Stepping	238.4 (65.8)	228.3 (48.5)	5.3 (−8.6, 19.1)
Step count			
All steps	292 (68)	281 (76)	4.9 (−10.9, 20.7)

* calculated as (activPAL4 − activPAL3)/activPAL3 as a %. LLOA/ULOA= lower/upper limits of agreement. ** very small amounts of sitting time for non-standardised activities.

**Table 3:** Confusion matrix for time spent on standardised and non-standardised activities between categorisation of sitting, standing, and stepping time (s) by the activPAL3^TM^ and activPAL4^TM^ (mean and range of values). [% of time (range) of activPAL3 ^TM^ classification split by activPAL4 ^TM^ classification].

**Standardised Activities**				
			ActivPAL3^TM^	
	Category of physical behaviour	Sitting	Standing	Stepping
ActivPAL4^TM^	Sitting	531.4 (450.5, 551.9) [100.0 (99.9, 100.0)]	7.1 (0.1, 86.8) [5.0 (0.1, 55.1)]	0.0 (0.0, 0.0) [0.0 (0.0, 0.0)]
	Standing	0.2 (0.0, 0.5) [0.0 (0.0, 0.1)]	70.3 (64.4, 77.5) [92.0 (44.2, 99.9)]	1.1 (0.1, 3.4) [1.0 (0.1, 3.0)]
	Stepping	0.0 (0.0, 0.0) [0.0 (0.0, 0.0)]	2.7 (0.0, 20.7) [3.1 (0.0, 21.0)]	112.9 (86.6, 121.6) [99.0 (97.0, 99.9)]
**Non-Standardised Activities**				
			ActivPAL3^TM^	
	Category of physical behaviour	Sitting	Standing	Stepping
ActivPAL4^TM^	Sitting	20.8 (2.3, 46.2) [96.4 (87.2, 100.0)]	1.0 (0.1, 6.9) [1.0 (0.2, 7.4)]	1.2 (0.0, 6.4) [0.5 (0.0, 2.5)]
	Standing	0.8 (0.0, 3.7) [3.4 (0.0, 11.0)]	87.4 (12.0, 162.1) [75.5 (54.4, 88.3)]	13.1 (5.8, 37.5) [5.7 (3.4, 15.3)]
	Stepping	0.0 (0.0, 0.6) [0.1 (0.0, 1.8)]	24.4 (16.1, 47.1) [23.5 (11.4, 44.3)]	214.0 (137.3, 322.1) [93.8 (84.7, 96.0)]

**Table 4:** Laboratory-based standardised and non-standardised activities (*n* = 13) second-by-second posture agreement, sensitivity, specificity, positive predictive value (PPV), and negative predictive value (NPV) between activPAL4^TM^ and activPAL3^TM^ with reference as activPAL3^TM^. Outcomes given as; mean (SD) (range).

**Standardised Activities**				
Agreement (%)				
Sitting/standing/stepping	98.4 (3.4) (87.6, 99.9)			
	Sensitivity (%)	Specificity (%)	PPV (%)	NPV (%)
Sitting	100.0 (0.0) (99.9, 100.0)	97.3 (8.6) (68.6, 99.9)	98.7 (4.5) (83.8, 100.0)	99.9 (0.1) (99.7, 100.0)
Standing	92.0 (15.4) (44.2, 99.9)	99.8 (0.1) (99.5, 100.0)	98.2 (1.2) (95.3, 99.6)	98.5 (3.7) (86.6, 100.0)
Stepping	99.0 (0.8) (97.0, 99.9)	99.6 (0.9) (96.8, 100.0)	97.5 (5.2) (80.7, 100.0)	98.5 (3.7) (99.4, 100.0)
**Non-Standardised Activities**				
Agreement (%)				
Sitting/standing/stepping	88.8 (3.0) (83.1, 91.9)			
	Sensitivity (%)	Specificity (%)	PPV (%)	NPV (%)
Sitting	96.4 (3.3) (87.2, 100.0)	99.4 (0.8) (97.2, 100.0)	86.0 (20.9) (25.3, 98.8)	99.8 (0.3) (98.8, 100.0)
Standing	75.7 (10.5) (54.4, 88.3)	94.4 (2.8) (85.7, 96.0)	84.1 (10.0) (61.2, 95.4)	90.0 (4.3) (80.2, 94.7)
Stepping	93.8 (3.1) (84.7, 96.0)	79.8 (10.0) (58.8, 90.1)	89.3 (4.9) (79.7, 94.7)	86.1 (9.5) (63.8, 96.2)

**Table 5:** Full 24 h mean values of activity categorisation (sitting/lying, standing, stepping), sit-to-stand transitions, and step count with Bland–Altman percentage mean differences between activPAL4^TM^ and activPAL3^TM^.

Measure (*n* = 15)	ActivPAL4^TM^ Mean (SD)	ActivPAL3^TM^ Mean (SD)	Percentage Mean Difference (LLOA, ULOA) (%) *
Full 24 h days			
Duration (mins/day)			
Sitting/lying	1158.74 (93.4)	1148.8 (88.7)	0.9 (−4.1, 5.8)
Standing	174.9 (80.7)	188.1 (80.2)	−7.6 (−25.5, 10.3)
Stepping	106.4 (38.7)	103.1 (36.3)	1.7 (−13.2, 16.6)
Transitions (transitions/day)			
Sit-to-stand transitions	184 (77)	181 (72)	2.1 (−12.9, 17.0)
Step count (steps/day)			
All steps	8040 (2970)	7762 (2821)	2.2 (−13.1, 17.4)
Steps >100 steps/min cadence	1352 (757)	1336 (714)	6.6 (−38.8, 51.9)

* calculated as (activPAL4 − activPAL3)/activPAL3 as a percentage. LLOA, ULOA = lower and upper limits of agreement.

## Data Availability

To enquire about access to the data used to develop the outcome in this paper, please contact the lead author.

## Supplementary Materials

The following supporting information can be downloaded at https://www.mdpi.com/article/10.3390/s23218970/s1: Figure S1: Agreement plots between activPAL4^TM^ and activAPL3^TM^ sitting, standing, and stepping time outcomes. Figure S2: Agreement plots between activPAL4^TM^ and activAPL3^TM^ sitting, standing, and stepping time outcomes with outliers removed. Table S1 [For comparison with Table 2 of manuscript] Laboratory-based mean outcomes with two participants removed. Table S2: (For comparison with Table 5 of manuscript) Free-living data analysis with one participant removed.
