# Supplementary material for: Agreement of Two Physical Behaviour Monitors for Characterising Posture and Stepping in Children Aged 6–12 Years
Source: Sensors (Basel). 2023 Nov 4;23(21):8970. doi: 10.3390/s23218970 (PMC10648043; doi:10.3390/s23218970)
Supplement: Supplementary file 1 [file sensors-23-08970-s001.zip › sensors-2622062-supplementary.pdf]

Figure S1. Agreement plots between activPAL4™ and activAPL3™ sitting, standing, and stepping time outcomes. Outcomes are illustrated as percentage difference against mean. Mean percentage difference (short dashed line) and upper and lower limits of agreement (long dashed lines) are illustrated.

Standardised activity agreement (time in seconds)

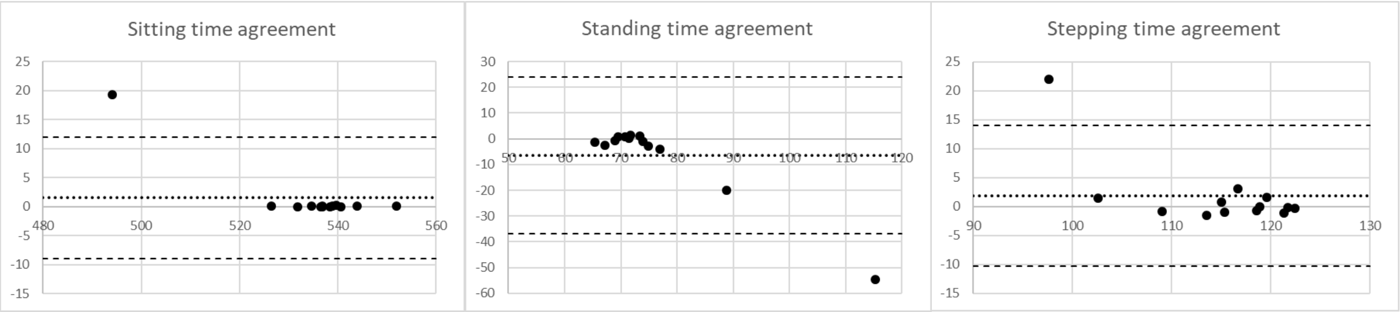


Non-standardised activity agreement (time in seconds)

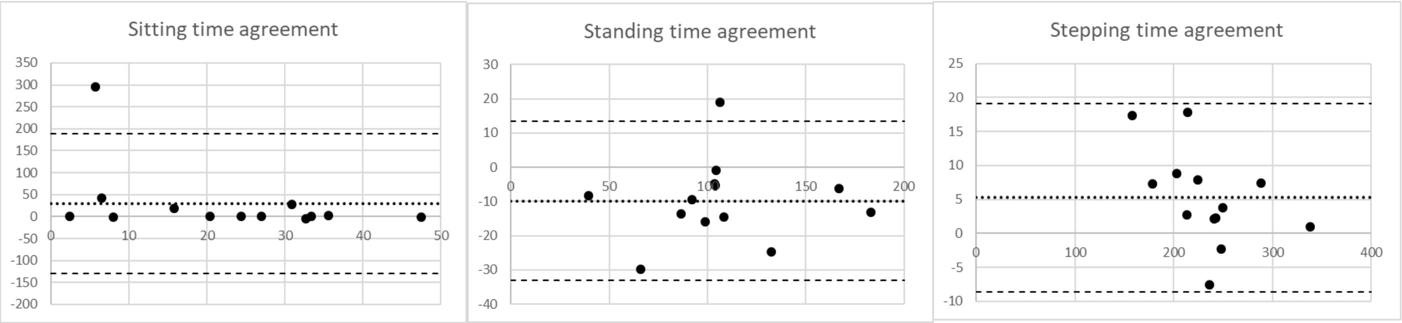


Step count agreement, standardised and non-standardised activities

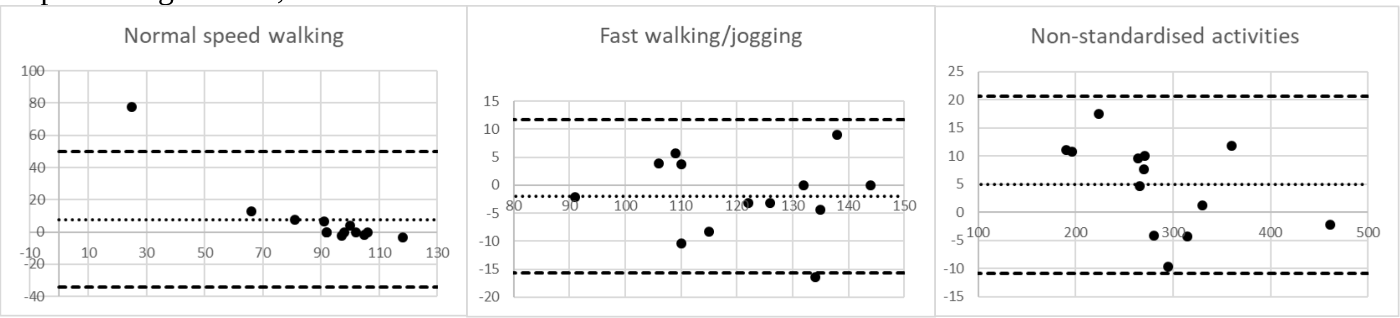

Free-living outcome agreement (time in minutes)

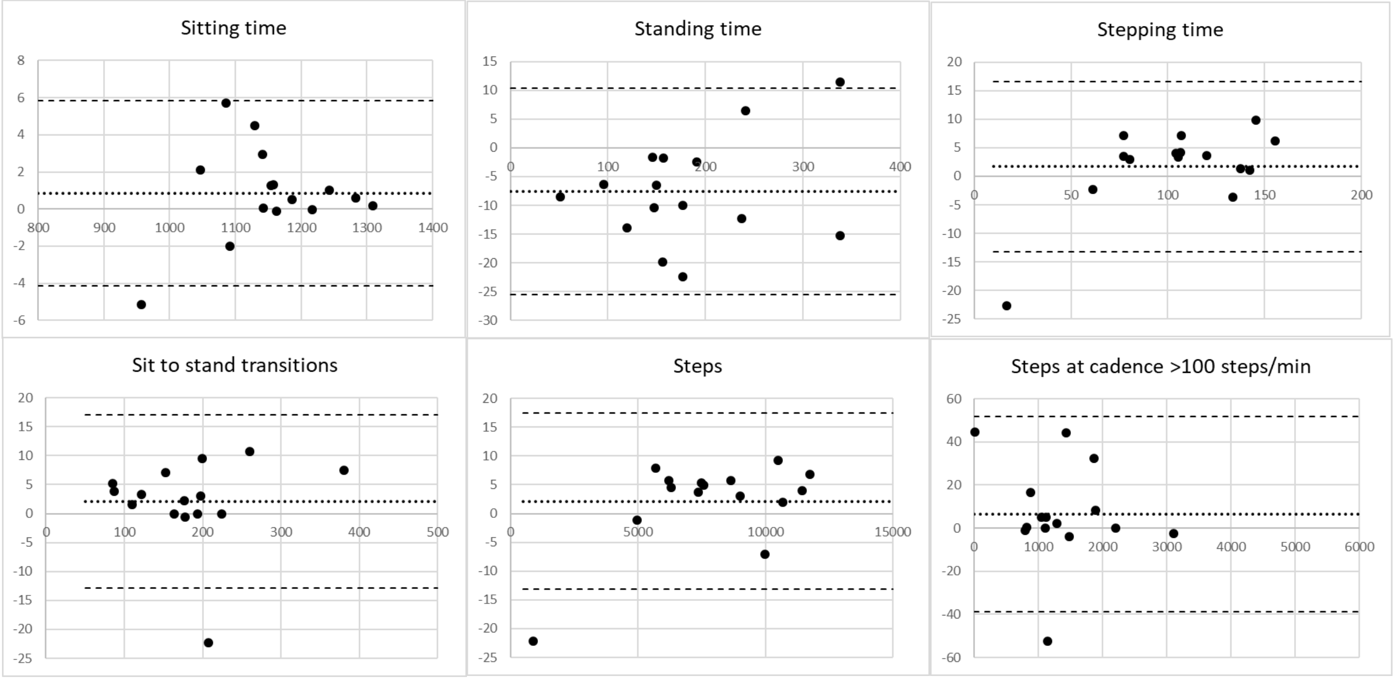

Figure S2. Agreement plots between activPAL4$^{TM}$ and activAPL3$^{TM}$ sitting, standing, and stepping time outcomes with outliers removed (two participants removed from laboratory data (one very slow stepping, one sitting with thigh at inclined angle) and one participant removed from free-living data (very slow stepping)). Outcomes are illustrated as percentage difference against mean. Mean percentage difference (short dashed line) and upper and lower limits of agreement (long dashed lines) are illustrated.

Standardised activity agreement (time in seconds)

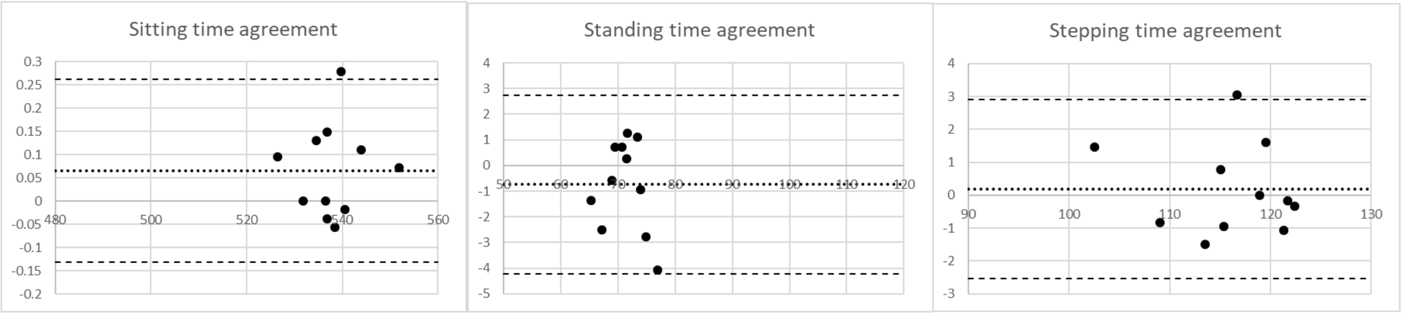


Non-standardised activity agreement (time in seconds)

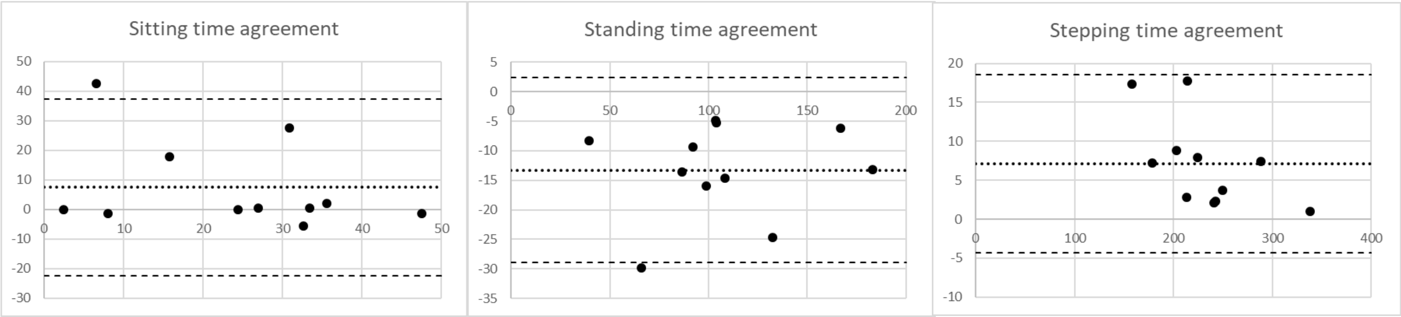

Step count agreement, standardised and non-standardised activities

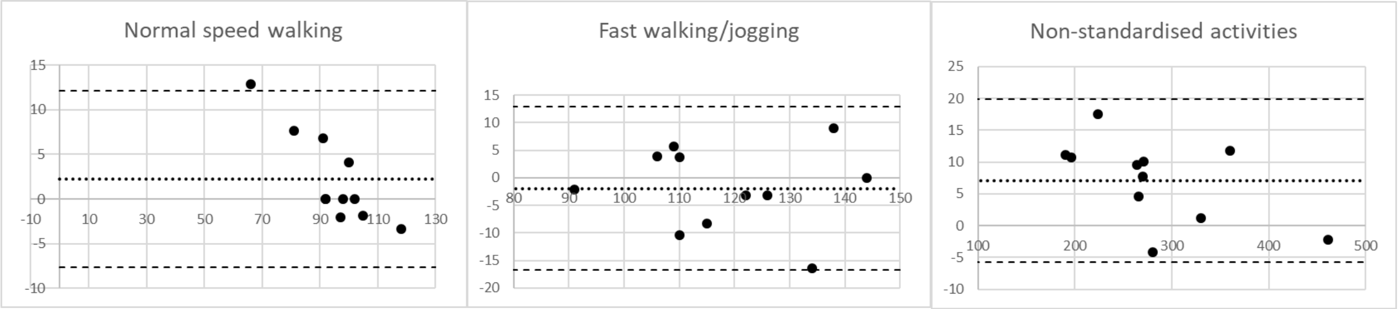


Free-living outcome agreement (time in minutes)

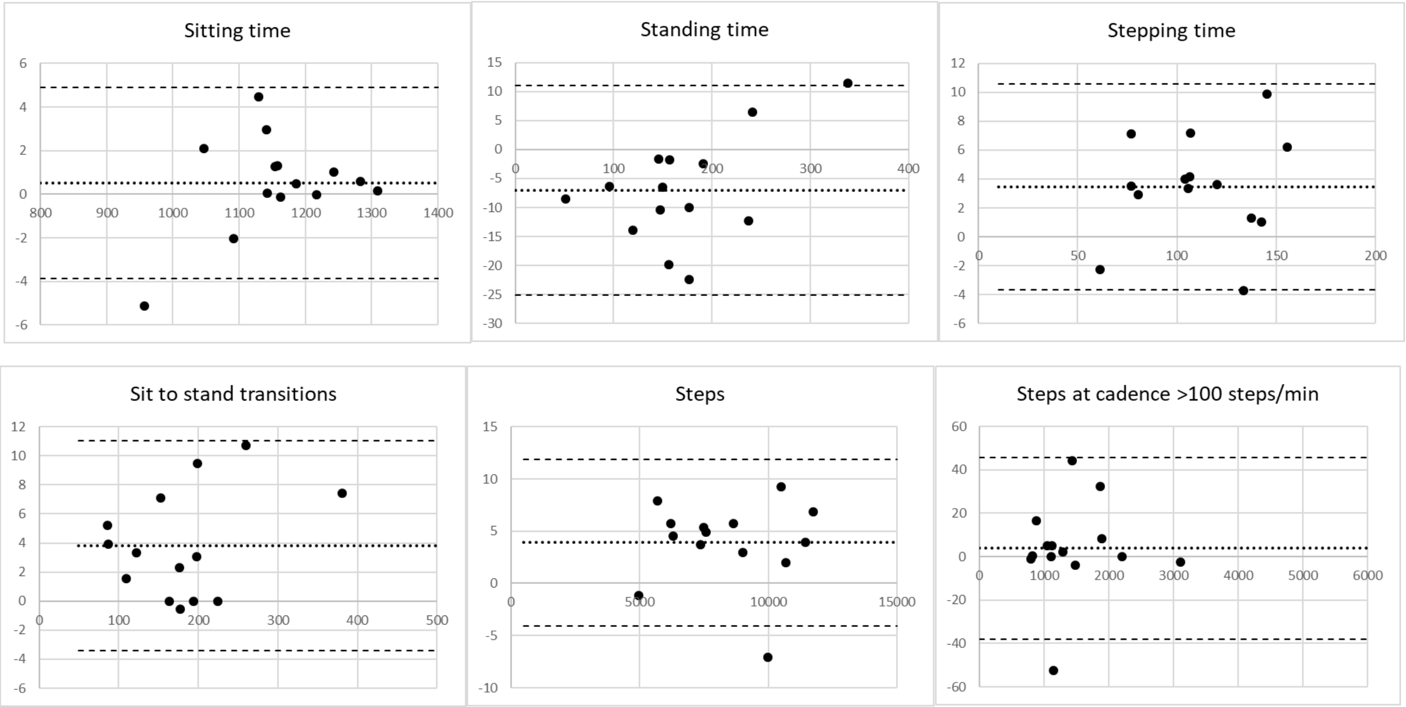

Table S1. [For comparison with Table 2 of manuscript] Laboratory-based mean outcomes with two participants removed (one very slow stepping, one sitting with thigh at inclined angle) with Bland–Altman percentage mean differences across standardised and non-standardised activities.

| Measure (n=11) | activPAL4™ Mean (SD) | activPAL3™ Mean (SD) | Percentage mean difference (LLOA, ULOA) (%)* |
|---|---|---|---|
| Standardised activities | | | |
| Duration (s) | | | |
| Sitting | 538.6 (6.8) | 538.2 (6.8) | 0.1 (-0.1,0.3) |
| Standing | 70.9 (3.3) | 71.4 (3.7) | -0.7 (-4.2,2.7) |
| Stepping | 116.1 (5.9) | 115.9 (6.2) | 0.2 (-2.5,2.9) |
| Step count | | | |
| Normal walking | 95 (12) | 94 (15) | 2.2 (-7.7,12.1) |
| Fast walking/jogging | 117 (16) | 120 (17) | -1.9 (-16.8,12.9) |
| Non-standardised activities | | | |
| Duration (s) | | | |
| Sitting | 24.5 (14.1) | 23.5 (14.3) | 7.5 (-22.3,37.7) |
| Standing | 114.9 (43.6) | 99.7 (39.4) | -13.3 (-28.9,2.4) |
| Stepping | 224.6 (52.2) | 238.8 (48.0) | 7.1 (-4.3,18.5) |
| Step count | | | |
| All steps | 291 (75) | 275 (82) | 7.1 (-5.7,19.9) |

*calculated as (activPAL4 - activPAL3)/activPAL3 as a %. LLOA, ULOA = lower and upper limits of agreement.

Table S2. [For comparison with Table 5 of manuscript] Free-living data analysis with one participant removed (low step volume and slow stepping). Full 24-hour mean values of activity categorisation (sitting/lying, standing, stepping), sit-to-stand transitions, and step count with Bland–Altman percentage mean differences between activPAL4™ and activPAL3™.

| Measure (n=14) | activPAL4™ Mean (SD) | activPAL3™ Mean (SD) | Percentage mean difference (LLOA, ULOA) (%)* |
|---|---|---|---|
| Full 24-hour days | | | |
| Duration (mins/day) | | | |
| Sitting/lying | 1161.8 (96.1) | 1155.5 (88.1) | 0.5 (-3.9,4.9) |
| Standing | 165.2 (74.2) | 175.4 (65.7) | -7.0 (-25.1,11.0) |
| Stepping | 112.9 (30.3) | 109.1 (28.9) | 3.4 (-3.7,10.6) |
| Transitions (transitions/day) | | | |
| Sit-to-stand transitions | 184 (80) | 177 (73) | 3.8 (-3.4,11.1) |
| Step count (steps/day) | | | |
| All steps | 8559 (2268) | 8245 (2190) | 3.9 (-4.1,11.98) |
| Steps >100 steps/min cadence | 1448 (685) | 1431 (634) | 3.9 (-38.1,45.8) |

*calculated as (ActivPAL4 - ActivPAL3)/ActivPAL3 as a %. LLOA, ULOA = lower and upper limits of agreement.
